# Household needs among wildfire survivors in the 2017 Northern California wildfires

**DOI:** 10.1088/2752-5309/ad951c

**Published:** 2025-01-10

**Authors:** Mitchell Snyder, Mira Miles, Irva Hertz-Picciotto, Kathryn C Conlon

**Affiliations:** 1 Geography Graduate Group, University of California, Davis, United States of America; 2 Department of Public Health Sciences, School of Medicine, University of California, Davis, United States of America

**Keywords:** wildfire, recovery, needs assessment, impacts

## Abstract

Wildfires are impacting communities globally, with California wildfires often breaking records of size and destructiveness. Knowing how communities are affected by these wildfires is vital to understanding recovery. We sought to identify impacted communities’ post-wildfire needs and characterize how those needs change over time. The WHAT-Now study deployed a survey that was made publicly available for communities affected by the October 2017 Northern California wildfires or the accompanying smoke at beginning approximately four months post-fire with the vast majority completed by nine months post-fire. Among other questions, the survey asked an adult household member to report on their households’ greatest need both one-week post-fire and at the time of survey. A total of 1461 households responded to these questions. Households reported many types of needs, with 154 responses that did not directly name needs but rather described how their households had been affected, which we classified as impacts. Four major themes were identified: physical, health, air, and information, each representing an array of varied specific needs or impacts. Physical needs (e.g. housing, food) were the most common (cited by more than 50% during the fires and about a third at the time of survey). The need for clean air was strong during the fires, but not months later, at the time of survey. In contrast, health needs were reported by a quarter of households during the fires. Needs that were reported at both times were categorized as ‘persistent’, and there were more persistent mental health needs over time compared to other health themes. Understanding the needs and impacts that arise during wildfires, their diversity and duration, and how they change over time is crucial to identifying types of assistance that are most needed during recovery efforts and when they are needed. Results presented here along with other wildfire needs assessments can be utilized to improve disaster preparedness, including for wildfire recovery.

## 1. Introduction

Climate change exacerbates the threat of wildfires globally, including in California, where prolonged severe drought, high ambient temperatures, and dead or dried forested areas associated with a changing climate foster the emergence and spread of wildfires [1]. Climate projections indicate that Californian wildfires will increase in intensity, frequency, and burn area in the absence of substantial disruption to the growing climate crisis [1–4]. Also contributing to wildfire threats, population centers in the United States, and particularly in Northern California, have grown substantially into surrounding wildland and vegetated areas known as the wildland urban interface (WUI). California has the nation’s highest number of WUI housing, consisting of more than 45% of all housing units in the state [5]. This corresponds to over 5 million households falling within WUI boundaries, located mostly along the Sierra Nevada mountains and in coastal areas [5–9]. Record-breaking wildfires have affected increasingly larger numbers of people across the American West [2, 6, 7, 10] and as more people move to the WUI, they put themselves at higher wildfire risk, given the historic trends and future predictions for a drier, warmer climate. Both in California and throughout the American West, this volatile combination of a drier, warmer climate and increasing WUI development means increasing numbers of people face unprecedented risks to their homes, their pets, their health, and their lives. Threats from extreme wildfire events extend globally, with impacts on countries such as Australia [11, 12], Greece [13, 14], and Canada [15]. Projections are dire for increased future wildfire risks across the globe [16–19].

**Figure 1.**
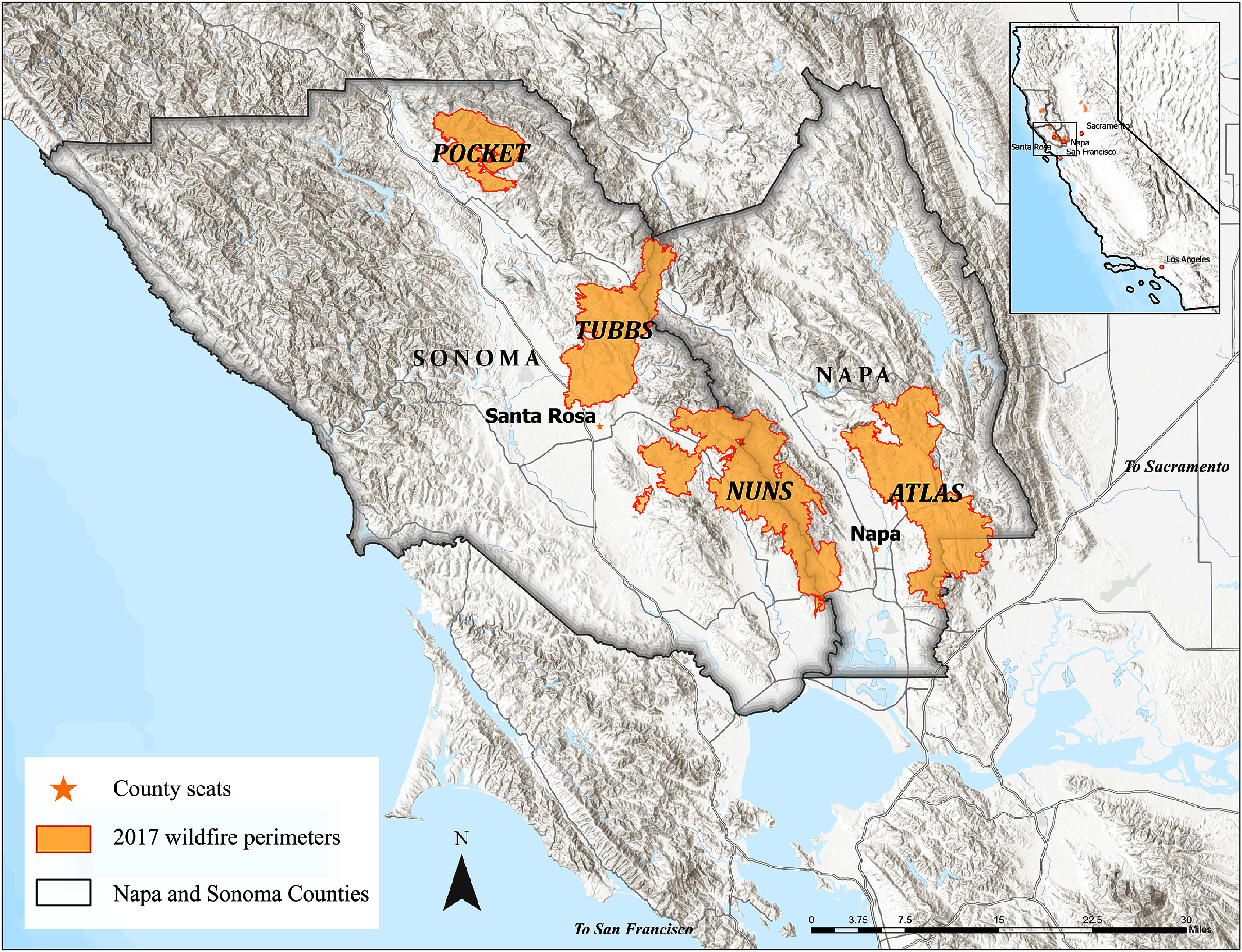
Burn area map in Napa and Sonoma Counties. The highlighted areas show the perimeters of the October 2017 North Bay Atlas, Nuns, Pocket, and Tubbs wildfires.

An unprecedented multi-wildfire event in the fall of 2017 struck WUI areas and marked the beginning of a new era in California wildfires in terms of their magnitude and destructiveness. Beginning on the night of 8 October 2017, a complex of wildfires burned across Northern California for approximately three weeks. High wind conditions, unique topographic features, and poorly maintained power lines contributed to the rapid spread of the wildfires, the largest of which occurred in Napa and Sonoma counties north of San Francisco (figure 1) [20, 21]. Collectively, these wildfires spanned eight counties, caused 44 fatalities, burned over 240 000 acres of land and destroyed nearly 9000 structures, including entire neighborhoods [22]. The Tubbs, Atlas, Nuns, and Pocket Fires were among the most destructive, displacing more than 90 000 people across Napa and Sonoma Counties [23]. A state of emergency was declared on 9 October 2017. Over $11 billion in insured damages were reported across the eight counties [21]. At the time, these were the most destructive and costly wildfires in California history. Those who evacuated had to rely on emergency shelters, family, friends, and other temporary housing options [8]. Households that did not evacuate likely remained in nearby communities that experienced resource strain because of the wildfires. When disasters displace communities, affected populations are faced with immediate and long-term economic, health, and social impacts [24].

Wildfire-affected communities are immediately faced with material losses such as basic needs like food, clothing, and secure housing. Previous research has found that lower resourced populations are less financially able to recover from the losses of a disaster [24, 25]. It is common for communities to be without functioning utilities such as gas, water, electricity, and phone service shortly after a disaster, which may impede community recovery. Direct health impacts such as injuries [26–28], exacerbation of existing chronic illness [29, 30], and mental health outcomes [31] are common among communities that experience wildfire. In this context, the extent to which a community has access to financial resources can influence the speed and scope of how community services are restored. Impacted households may lose their home to a wildfire or may be evacuated. Displacement creates a need for short- and long-term housing. Amidst an ongoing housing crisis in California, population displacement resulting from wildfires stresses a fragile housing economy and introduces challenges for displaced individuals as they migrate or compete for limited housing resources [32]. The housing recovery process of displaced populations highlights inequities across different socioeconomic groups affected by wildfires, with higher-income households more likely to be able to migrate or rebuild to resolve their housing needs in the short-term [33]. Rural communities affected by wildfires may have minimal personal and public resources available to them to assist with recovery, perpetuating or exacerbating the health inequities and social determinants of health that put these communities at risk [34]. Challenges to economic stability [34, 35], physical environment, safety [36, 37], and health care systems compromise the health and well-being of wildfire impacted communities. To address the diverse and dynamic needs that arise after wildfires and other disasters, researchers and government officials use a variety of tools such as needs assessments to identify and develop strategies to address needs.

Existing literature on community needs assessments following natural disasters is limited. Needs assessments in disaster contexts can offer insight into the priority needs of the affected community and can inform emergency response organizations for strategic planning of what response and support measures should be deployed [21]. While there have been studies on specific disaster outcomes such as mental health effects and the role of community cohesion and resilience, there is a paucity of research on the broad needs surrounding communities impacted by wildfire. Social science studies on post-wildfire communities offer insight on effective public communication methods such as tailored outreach and messaging, which can be accomplished by better understanding unique household experiences with wildfire and post-wildfire risks [38, 39]. Understanding social dynamics between residents affected by wildfires, government officials, and others involved in wildfire management or recovery efforts in local contexts can support wildfire adaptation and mitigation approaches [40, 41].

This research is part of a larger study that explores the impacts of the 2017 Northern California wildfires, called ‘***W***ildfires and ***H***ealth: ***A***ssessing the ***T***oll in ***No***rthern ***Ca***lifornia Study’, abbreviated as the: WHAT Now, CA? Study [42]. WHAT Now, CA is a longitudinal study that enrolled households affected by one or more of California’s wildfires beginning in 2017. Data collected through an online Qualtrics survey covered household members’ experiences during and after the wildfires, unmet needs, health symptoms and recovery [42]. In the project reported here, we sought to identify impacted communities’ post-wildfire needs and characterize how those needs change over subsequent months. The findings from this research provide information that can be useful to county health departments, preparedness and response agencies, medical providers, policy makers, and non-profit organizations to prepare for and respond to community needs during and following future wildfires.

## 2. Methods

Beginning in late January 2018, the WHAT Now, CA online survey was made available, with the target population being households in counties impacted by the 2017 Northern California wildfires or by the smoke produced by those wildfires. Eligible respondents included anyone over the age of 18 in households located within those counties. There was no further restriction, as the goal was to capture the experiences of a wide swath of persons in those counties. The survey was informed by previously deployed post-disaster survey instruments, by reported experiences in the media (social and traditional), and by consultations with Napa and Sonoma County Health Department personnel. Particularly influential for question formulation and content was the feedback we received from Napa and Sonoma County epidemiologists and public health officers. Based on their input, we incorporated two open-ended questions related to needs during and in the aftermath of the fires. These questions about unmet needs created by the fires are the basis for the current project. The draft survey was pilot-tested for ambiguity, appropriateness of language, and length. The consent form and survey were programmed into electronic format and reviewed for accuracy of skip patterns. The English version, along with Spanish translation completed by a bilingual/bicultural (Mexican origin) University of California Davis staff person and reviewed by two other native Spanish speakers at the Napa County Public Health, were both approved by the University of California, Davis Institutional Review Board.

We recruited survey participants from California counties affected by wildfires and included counties affected only by the smoke from those fires. We sought to include Northern California counties that may have included persons not exposed to the fire or smoke in order to capture exposure contrasts. Recruitment occurred using various media, including print, radio, and substantial social media efforts to reach a broad public. Social media posts, particularly in wildfire-specific Facebook groups, were the most successful recruiting tool, as those postings led to the largest proportion of respondents. Coverage in local (i.e. county, city) newspapers, radio interviews, and word-of-mouth bolstered the recruitment. A large convenience sample was enrolled, in which 2208 households participated. Respondents with reported needs predominately came from the eight Northern California counties that experienced wildfires starting on 8 October 2017: Butte, Lake, Mendocino, Napa, Nevada, Solano, Sonoma, and Yuba counties.

Survey respondents answered questions on behalf of their household. Of the 2208 households that participated, 1461 answered one or both of the key questions: (1) what was your household’s greatest need one week after the wildfires?, (2) What is your household’s greatest need currently? The vast majority of respondents completed the survey in the period from four through nine months post-wildfire (late January through June/July 2018). As a result, ‘currently’ varied according to when the respondent completed the survey, with the average time from the wildfire at five and a half months, reflecting that most households responded in the first four months the survey was available. Closed-ended survey questions, which are not analyzed here, captured evacuation experiences during the wildfires, sociodemographic characteristics, losses (e.g. home, source of income, family members or close friends, pets) newly occurring health symptoms—both physical and mental—as well as pre-existing conditions before the wildfires [42].

### 2.1. Coding methodology

Qualitative coding was used to characterize and analyze the responses to the open-ended questions. Survey responses were anonymized with randomly assigned, unique household identifiers. All responses were systematically categorized using a codebook developed by two coders. We used emergent coding methodology to identify common themes (hereafter referred to as major themes) and sub-themes. Given the temporal element of the survey questions, we identified two time periods: immediate, or needs which occurred in the week following the wildfire, and time-of-survey (ToS); however, we further classified responses that were reported by a given household for both immediate and ToS as a third category: persistent. ‘Persistent’ refers to needs and impacts reported for both time points, in which a household reported the same need for both key questions. Although both survey prompts asked for the greatest need (in the week following the wildfire and at the ToS), respondents varied in their responses. Some provided many needs, while others provided a single need. We coded all reported needs.

### 2.2. Thematic analysis

We developed a codebook that contained detailed inclusion and exclusion criteria for major themes and sub-themes (supplement B). This process was guided by prior research on intercoder reliability [43], qualitative disaster research [44], and relevant qualitative studies of health [45], including wildfire specific studies [37, 46]. Needs were coded into four major themes: physical, health, air, and information. Survey responses could contain one, many, or all of the major themes. Responses that detailed an impact were coded as such and differentiated from needs in the sub-theme categories. The branching relationships between major and sub-themes are presented in supplement A. We used intercoder reliability to measure agreement and consistency between coders categorizing qualitative data on a nominal scale [47, 48]. Intercoder reliability was established with multiple rounds (*n* = 5) of subsets of responses (*n* = 50 per round) coded independently by the two coders using the same codebook. Results were compared to identify discrepancies, which were then discussed until coders came to 100% agreement. The remainder of responses were then coded and an intercoder reliability of >80% agreement between coders was established and maintained. This process ensured high agreement and accuracy between coders.

The health theme was divided into physical health and mental health. Health-related responses were further delineated as either a need or an impact. The distinction between needs and impacts emerged upon reviewing survey responses that explicitly provided information about how a respondent was impacted (e.g. health effects) rather than their needs (e.g. resources). Health needs were defined as physical or psychological requirements for a person’s wellbeing, and health impacts were defined as physical or psychological effects on a person’s wellbeing.

## 3. Results

Of the 2208 total households that participated in the survey, 1461 households with reported needs answered one or both questions related to their greatest needs. Respondents included households that lost their homes to wildfires; households that did not lose their housing to the wildfires, but did evacuate; those that were exposed to wildfire smoke but did not evacuate; and yet others that hosted families or friends that were directly affected. We used all the information provided by households that reported more than one need. We recorded 2491 needs across the 1461 household with reported needs. The following quote illustrates how a respondent identified more than one type of need: *Adequate rest, emergency equipment/masks, gasoline, water, food. I had to sleep in my car as there was nowhere else to go. I thought I was safe, but on the third night I awoke in the middle of the night and with the flashlight I could see my car was full of floating ash. I had been sleeping, breathing all this in for days. I still had no mask. I went to two hospitals and they were overrun and had [run] out of masks, as did all the stores.*

We identify physical needs (e.g. gasoline, food, water, shelter), health needs (e.g. adequate rest), air needs (e.g. floating ash and need for masks), and information needs (e.g. a safe place to go for sleep and supplies, warnings about air quality).

Over 30% of total households (*n* = 747) either did not report having needs or did not answer the open-ended questions and were therefore excluded from this analysis. More than half (57.5%) of household with reported needs answered both questions related to their greatest needs, while only 9% answered a single question. Table 1 contains the descriptive statistics of these household groups. Demographically, in households with reported needs, respondents were predominantly white (89.9%), or female (80.9%), and for three quarters of those households, a member of the household was the homeowner (73.2%). We compared demographics of the WHAT-Now, CA? Study participants in each of the three top counties represented in our sample with the demographics of the corresponding county population in 2017 [49]. Females were far more likely to be the respondents, which likely is attributed to the greater use of Facebook, one of our most successful modes of recruitment, by females than males, particularly at that time, when it was a relatively new platform. Other differences were a slightly higher representation of whites and persons with more than a high school education in each of the three counties, and substantial differences for bachelor’s or higher education and home ownership. It should be noted that the survey participants were more likely to have lost their homes than the general population in those counties, and that although fires can in some regions be considered ‘random’ in their destruction, the residents in WUI areas had greater losses of home and property in these 2017 fires and on average were much wealthier, while those of lower socioeconomic levels were less likely to lose their homes. Thus, the skewness in our study sample actually reflected the skewed acute wildfire impacts across different communities. Future analyses of long-term recovery may reveal patterns of a different type, with greater impacts in the lower resource communities [49]. Also noteworthy is that Sonoma County households made up 74.9% of households with reported needs, with 1049 of 1461 households.

**Table 1.** Descriptive summary statistics of total household with reported needs and households with reported needs. Discrepancies between reported demographics and population sizes are due to not all survey respondents answering both needs and demographics questions.

	Total households (*n* = 2208)	Households with a reported need (*n* = 1461)	Households without a reported need (*n* = 747)
	*N* (%)	*N* (%)	*N* (%)
Sex			
Female	1571 (71.2%)	1182 (80.9%)	389 (52.1%)
Male	360 (16.3%)	234 (16%)	126 (16.9%)
Missingness	277 (12.5%)	45 (3.1%)	232 (31.1%)
Age (years)			
18–35	360 (16.3%)	240 (16.4%)	120 (16.1%)
36–45	316 (14.3%)	227 (15.5%)	89 (11.9%)
46–55	355 (16.1%)	255 (17.5%)	100 (13.4%)
56–64	444 (20.1%)	339 (23.2%)	105 (14.1%)
65 or older	393 (17.8%)	315 (21.6%)	78 (10.4%)
Missingness	340 (15.4%)	85 (5.8%)	255 (34.1%)
Race			
White	1631 (88.8%)	1220 (89.8%)	411 (86.2%)
African America/Black	8 (0.4%)	6 (0.4%)	2 (0.4%)
Native American	16 (0.9%)	7 (0.5%)	9 (1.9%)
Asian/Pacific Islander	52 (2.8%)	30 (2.2%)	24 (4.4%)
Multiple races	90 (4.9%)	65 (4.8%)	25 (5.2%)
Other	39 (2.1%)	31 (2.3%)	8 (1.7%)
Missingness	372 (16.8%)	102 (7.0%)	270 (36.1%)
Educational level			
High school graduate or equivalent	63 (2.9%)	45 (3.1%)	18 (2.4%)
Some college, but no degree	361 (16.3%)	262 (17.9%)	99 (13.3%)
Trade or associate degree	274 (12.4%)	208 (14.2%)	66 (8.8%)
Bachelor degree	635 (28.8%)	467 (32.0%)	168 (22.5%)
Graduate degree	512 (23.2%)	388 (26.6%)	124 (16.6%)
Missingness	363 (16.4%)	91 (6.2%)	272 (36.4%)
Housing status			
Homeowner	1581 (73.2%)	1070 (73.2%)	511 (68.4%)
Renter	593 (26.9%)	377 (25.8%)	216 (28.9%)
Other	21 (1.0%)	11 (0.8%)	10 (1.3%)
Missingness	13 (0.6%)	3 (0.2%)	10 (1.3%)

**Figure 2.**
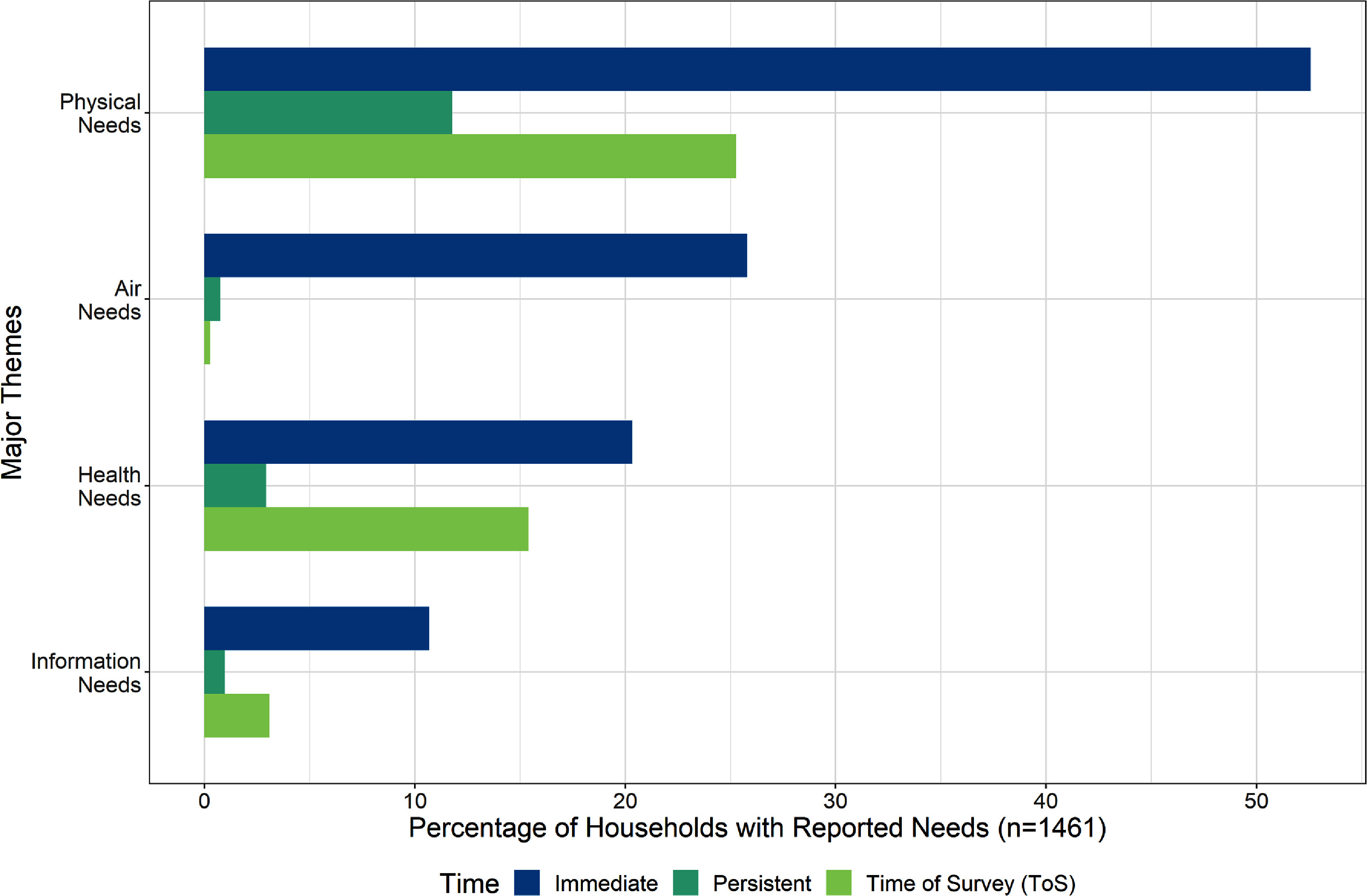
Major themes as a percentage of households with reported needs, at survey timepoints. Major themes and their sub-themes are further defined in the codebook and tree diagram found in supplement A. Themes are not mutually exclusive; some households reported multiple themes. Timepoints are mutually exclusive.

Figure 2 presents four major themes—physical, health, air and information needs—from one or both time points. Physical needs predominated immediately following the wildfire and continued to be the most common at all time points. Air needs were the second most commonly reported at the immediate timepoint, but precipitously dropped for persistent and ToS periods. In contrast, the health theme, which included both physical and mental health needs, was the second most commonly reported to be persistent and newly reported at the ToS. Among households reporting greatest needs at the ToS, more than 25% and more than 15%, reported, respectively, physical needs and health needs that they did not report for the immediate period.

### 3.1. Physical needs

**Figure 3.**
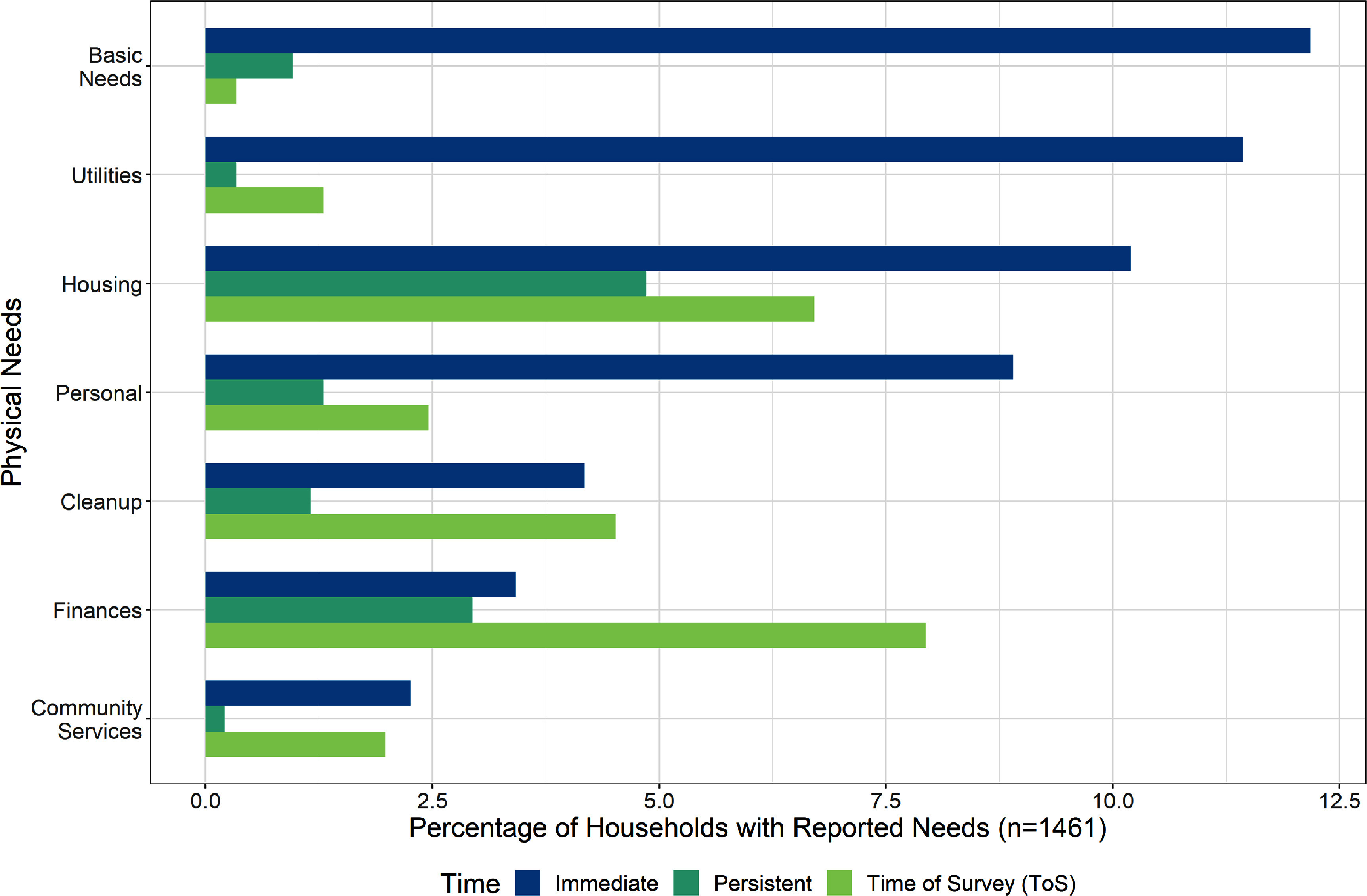
Physical needs themes as a percentage of households with reported needs, at survey timepoints. Sub-themes that comprise basic needs, utilities, housing, personal, cleanup, finances, and community services are further defined in the codebook and tree diagram found in supplement A. Themes are not mutually exclusive; some households reported multiple themes. Timepoints are mutually exclusive.

Physical needs were the most frequently identified survey responses. These are basic human needs like food, water, clothing, shelter, and other items that materially assist recovery, such as money, cleanup, or utilities (e.g. electricity, internet, gas, or cell phone service). Respondents frequently listed more than one physical need in their responses, particularly in the immediate timeframe. The top three immediate physical subthemes were: basic needs, utilities, and housing needs (figure 3). Personal needs, including hygiene and personal care items, were also commonly reported physical needs. Needs varied over time. In the immediate aftermath, basic needs and utilities were a priority, but waned as financial needs increased and housing needs persisted over the course of the survey period.

### 3.2. Health needs and impacts

**Figure 4.**
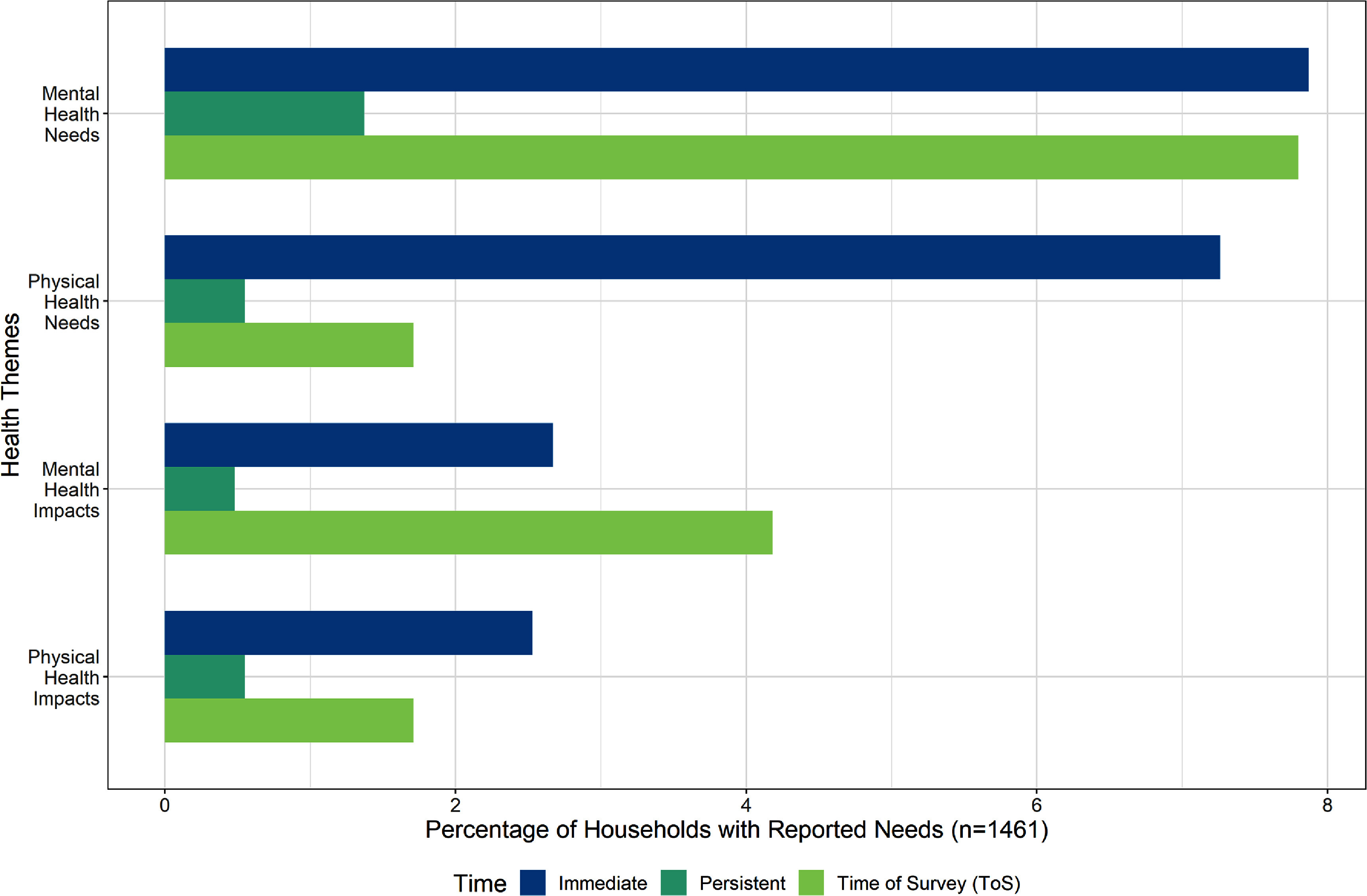
Major health themes as a percentage of households with reported needs, at survey timepoints. Themes are not mutually exclusive; some households identified multiple themes. Timepoints are mutually exclusive.

Health care and sustained health care access were challenges for many survey respondents in the weeks to months following the wildfires. Over 500 health-related responses were reported, spanning physical and mental health needs and impacts (figure 4). A key result of this study was that respondents answered the questions differently, some reporting health needs and others reporting the health impacts of the wildfires. We consider health needs to be physical or psychological requirements for a person’s wellbeing. For instance, a physical health need might be restored health or a sanitary home. Mental health needs include generalized support or explicit requests for professional services. Nearly four times as many households reported mental health needs either immediately following the wildfire or at the ToS as did households with persistent mental health needs (figure 4). Because each time point was mutually exclusive, mental health needs reported by households in the immediate time point are distinct from the households with mental health needs at the ToS. This was similar to other health needs, although the proportion with mental health needs at each of the three time categories was consistently higher than physical health needs, and physical and mental health impacts. The percent of respondent households with persistent mental health needs was nearly three-fold greater relative to households with persistent needs for other major themes. Although our survey prompt asked about needs, many responded with health impacts, such as respiratory health symptoms following the wildfires. Mental health impacts, in comparison, were responses that mentioned experiencing anxiety, trauma, or other generalized stresses. Physical health impacts were more frequently reported at immediate whereas mental health impacts were equally high at ToS versus the immediate period (figure 4).

#### 3.2.1. Physical health

We recorded 139 reports of physical health needs and 70 reports of physical health impacts and categorized them into subthemes. Physical health needs, such as medications, sleep and medical care, were listed four times more frequently in the immediate time point compared to the ToS. As one household stated:

*Getting pain medication … was our top priority and it was hard because Kaiser was shut down. We could not get through to doctors, nurses, or the pharmacy for many days … Medical professionals were not available because their own houses burned down, plus medical offices were closed due to fire damage or smoke…*

Respiratory health was a concern for the 70 households that reported physical health impacts. One respondent reported a need for ‘Medical care for the respiratory problems from the smoke… Even the dogs were coughing and sick from the smoke that enveloped us.’ Respiratory-related responses were often accompanied with worry about the potential toxicity and long-term impacts health effects of smoke and ash. Other impacts, reported by fewer than 0.5% of households, included cold and flu symptoms, or allergic reactions. Health needs, especially in the immediate time phase, often touched upon multiple major themes like air or information as pathways to health. Restored health and lingering respiratory health impacts were also front of mind for respondents at ToS, with twice as many households (<1%) reporting restored health as a need months post wildfire as compared with the immediate phase (<0.5%) one week after the fires. The co-reporting of major themes highlights how health was often a component alongside other major themes.

#### 3.2.2. Mental health

We recorded 249 mental health needs and 107 mental health impacts across all households with reported needs. Unlike the physical health need, mental health needs at both immediate and ToS time points were reported by an equal number of households, (figure 4), these needs and impacts were exclusive across time points, so that the households at the ToS are not the same households with immediate needs. Trauma, a sub-theme, was reported by nearly three times as many households at ToS (*n* = 18) compared to the immediate phase (*n* = 6). Examples of immediate mental health needs include healing, peace of mind, support, and therapy. The majority of households with mental health impacts described a general impact, though others specifically mentioned trauma, anxiety, and other specific mental health impacts. Half of those with general mental health impact indicated some level of stress or worry because of the wildfire. Households that reported anxiety and trauma explicitly used those words, saying they were ‘healing from trauma’ or ‘had anxiety’. General mental health impacts did not have to explicitly mention mental health, but instead would describe stressful experiences or situations, such as: ‘We survived by jumping into our pool and watched everything burn around us.’ Other responses were literal, stating they needed: ‘mental health care. We were shaken up.’ Over half of mental health impacts were reported at the ToS, compared to 39 households with immediate mental health impacts. Top persistent impacts included trauma and anxiety, which underscores those households which reported persistent needs for therapy or professional counseling.

### 3.3. Air needs

**Figure 5.**
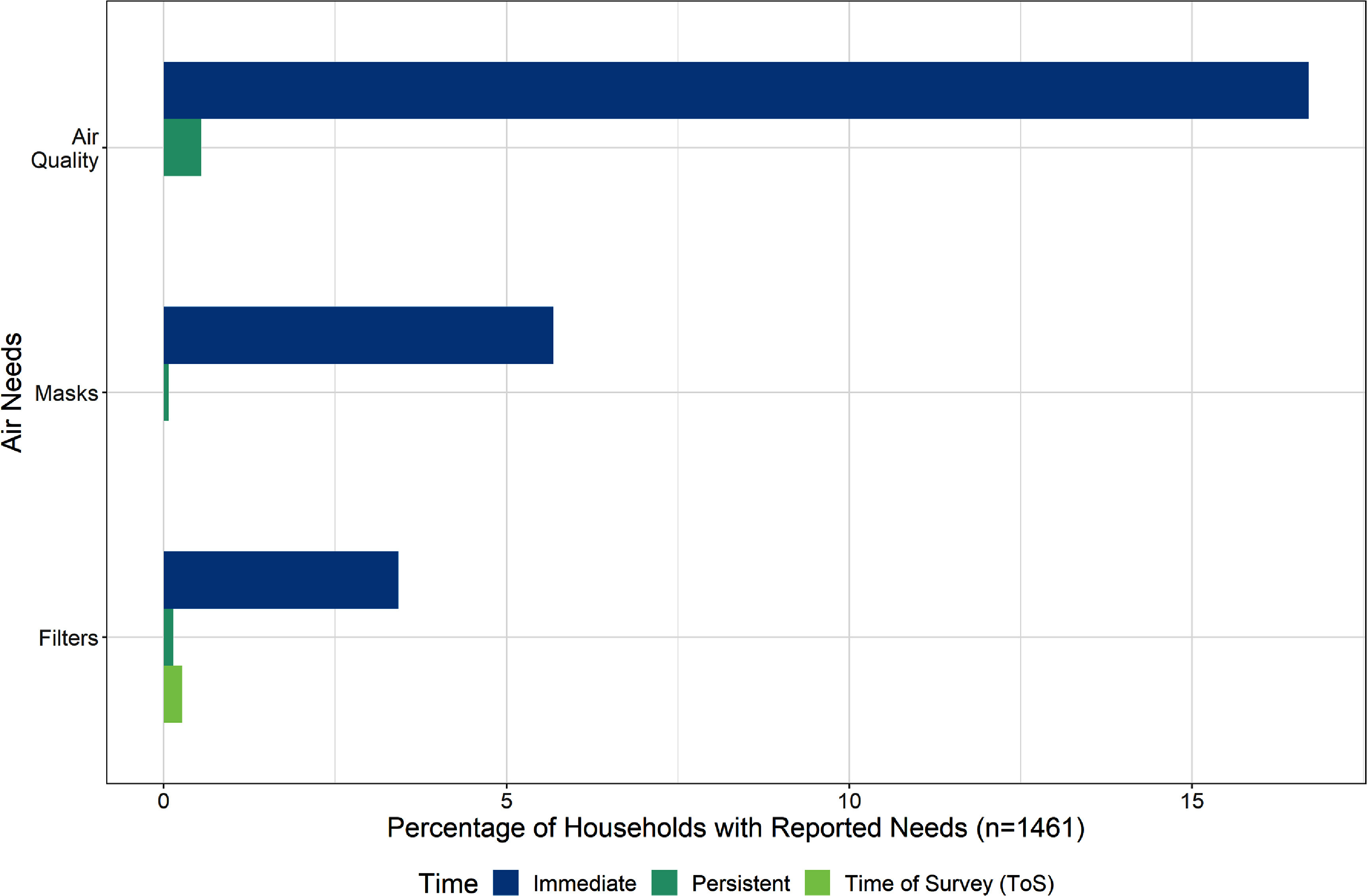
Themes pertaining to air needs as a percentage of households with reported needs, at survey timepoints. Themes are not mutually exclusive; some households reported multiple themes. Timepoints are mutually exclusive.

Air needs were identified by more than a quarter of respondents (*n* = 392) and can be characterized by reports desiring improved air quality (the majority) and access to masks and filters. Nearly all (96%) of these needs were identified in the immediate time point of the survey. As one respondent put it, ‘good air quality was the biggest concern’, which was the top air need. Masks and filters, which filter out harmful air pollutants from the air breathed, represented the remainder. There was a precipitous decline in air-related needs after the immediate time point, though a diminished few reported better air quality as persistent across the time periods (figure 5).

### 3.4. Information needs

**Figure 6.**
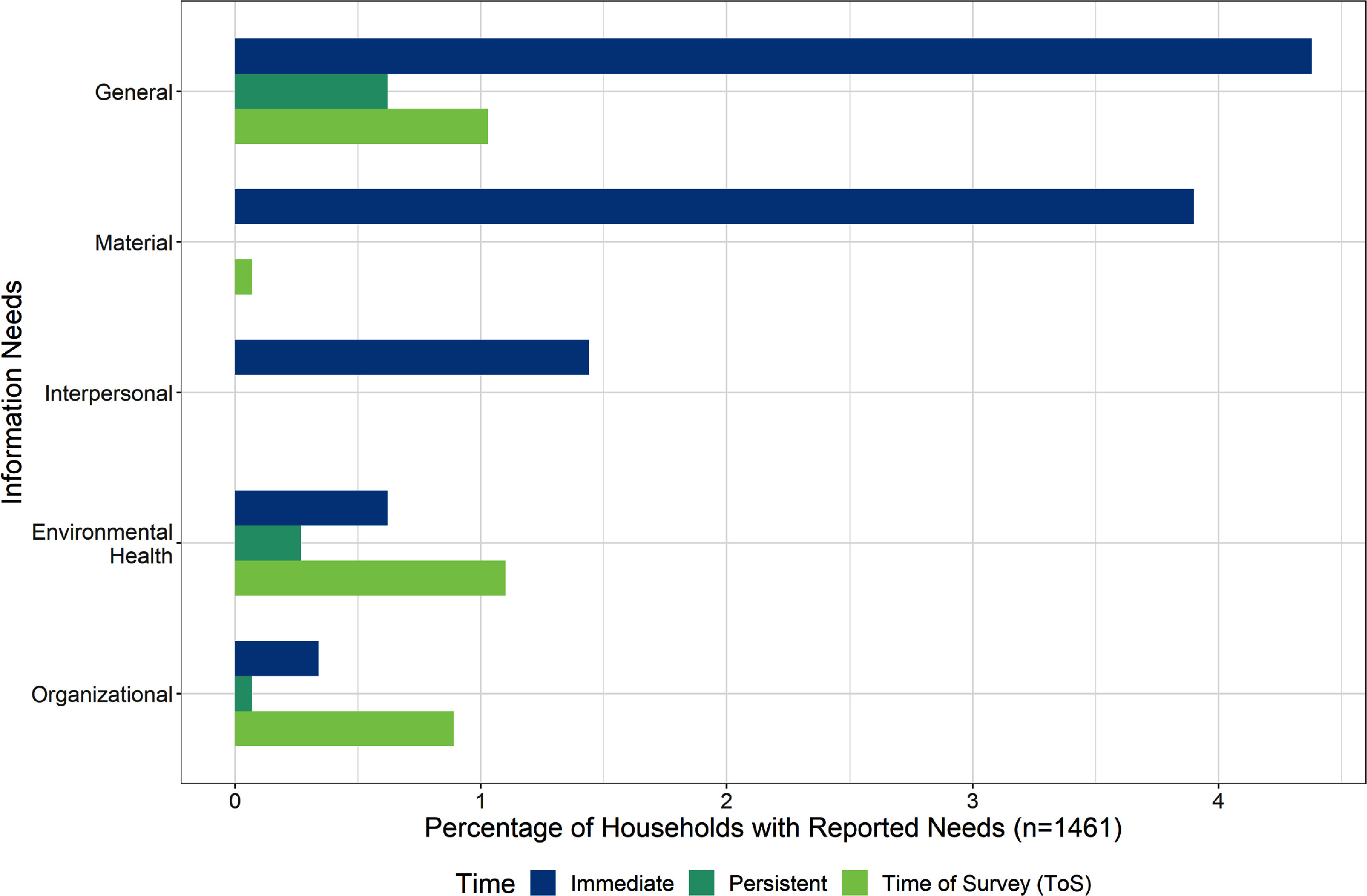
Themes pertaining to information needs as a percentage of households with reported needs, at survey timepoints. Sub-themes that comprise general, material, interpersonal, environmental health, and organizational needs are further defined in the codebook and tree diagram found in supplement A. Themes are not mutually exclusive; some households have multiple themes. Timepoints are mutually exclusive.

Information was a dynamic need for households, with 215 households reporting information-related needs across all time points (figure 6). Information needs represented numerous aspects of recovery, from the location and wellbeing of loved ones, to insurance paperwork, to the long-term health effects of smoke inhalation. Lack of current and reliable information was seen as an immediate need for many of the impacted households. As one respondent put it, their greatest need was:

*Information and reassurance. Many rumors flew about whether it was safe to return to the neighborhood (threats included the possible resurgence of fire, poor air quality, lawlessness/looting/shooting).*

The information needs were time-dependent: over 150 households reported the immediate need for information, which was more than three times the number of households needing information at ToS (figure 6). Among households with immediate information needs, approximately half did not elaborate on what information was lacking. Nearly 40% of immediate information needs specifically stated they were seeking material information relating to the status of the wildfire (e.g. wildfire containment, evacuation zones, personal safety, status of home). The fast-changing nature and unreliability of information was frustrating for some respondents. One wanted ‘accurate information especially as to fire situation at my home, often ALL [information] was wrong or non-existent or incomplete.’

Compared with the pressing immediate information needs at one week following the wildfires, persistent and ToS information needs were fairly general. They revolved around updating existing and circumstantial insurance, permitting, and rebuilding lines of communication between households and response agencies. More than a third of the 45 respondents at ToS reported concerns about the health effects of wildfires and sought information relating to wildfire smoke and environmental exposures (figure 6). Other prominent specific ToS informational needs included households’ need for reassurance, wanting to be better informed about preventing future wildfires, or protecting their properties. Whether respondents wanted improved health education, guidance on how to rebuild, or simply communication from a trusted source, these themes did vary over time.

## 4. Discussion

*I wish people had a better understanding of my needs: They gave us a case of [graham] crackers (I needed supplies, real food and personal hygiene supplies)*

Wildfires impact communities in numerous ways, as shown by the 1,461 households in Northern California that provided first-hand details about their needs and impacts following the 2017 wildfires. Responses highlight the multifaceted needs and impacts across three time periods: immediately following the wildfire, at the ToS (i.e. a few months after wildfires), and persistent (at both timepoints). Our findings illustrate the dynamic nature of household needs and post-disaster impacts. We distinguish between different types of needs by sorting them by major themes: physical, health, air, and information. Respondents answered on behalf of their household, with some responses containing a household’s sole greatest need while others providing multiple needs in a single response. More than half of respondents to the two questions reported needs during the immediate time point, and of these responses physical needs were the most common. Persistent physical needs for essentials like housing often included other major themes such as information or physical health. Our findings point to a pattern in which some respondents more commonly described how their health had been affected by the wildfire, rather than directly stating their health needs. Of 177 households reporting health impacts, the majority were related to mental health. Access to up-to-date information on wildfire status, air quality, safety of loved ones, and recovery-related processes were critical needs in the immediacy of the wildfires.

Physical needs often reflected the most noticeable or quantifiable losses in the wake of a disaster; survivors must prioritize basic needs such as food, water, shelter, and safety [50, 51]. Naturally, such housing-related needs were a leading concern, with long-term housing dominating the conversation. Considerations that ‘rents (housing stock) costs are spiraling out of control because of an already tight market before the fires’ complement previous research on post-disaster housing, linking home loss to prolonged recovery trajectories, especially for lower income households [34, 52]. Regional characteristics, like competitive housing markets, may impede both short- and long-term recovery. Wildfires in communities like Northern California, where housing is a premium, has exacerbated housing stock shortages, increase rental prices, and limit recovery trajectories [9, 53]. Residents and communities have been faced with inconsistent employment and economic insecurity, highlighting the ripple effect that wildfires and other disasters can have on recovering communities. These influences can create or perpetuate already existing financial and housing inequities [38]. Financial inequities can further drive barriers for individuals implementing disaster mitigation measures, such as additional insurance costs, that put lower income households at greater risk for losses from possible future fires [54]. One respondent wrote, ‘I was evicted from the residence because [my] landlord felt my job would be gone because of [the] fires.’ Although basic needs were the physical items identified by survey respondents, there were a host of associated needs that follow after a wildfire, such as housing access and financial support, that can indicate long-term post-fire recovery progress. These downstream effects are particularly challenging for vulnerable populations, as documented in Davies *et al* [34].

Post-wildfire health is a topic of growing interest, with recent studies linking wildfires to a range of impacts from smoke inhalation to mental health impacts [31, 52, 55, 56]. A quarter of the 249 households with mental health needs expressly reported ‘Peace of mind’ as a top need. Recent research by Grennan *et al* found that those exposed to wildfires displayed symptoms of post-traumatic stress disorder, anxiety, and depression [57]. Respondents to open-ended questions reported similar mental health impacts, including anxiety and trauma. Enduring needs related to mental health may be best addressed by leveraging existing community networks to facilitate cohesion. In the wake of the 2017 wildfires, the crisis counseling service California HOPE Sonoma was widely used by thousands of affected households for several years after the wildfires [58–60]. Respondents and many others engaged in informal information sharing networks, using ‘Several FB [Facebook] groups [to stay] on top of fire containment, etc, updates, and broadcast live updates from the Sheriff’s office.’ Long-term, unresolved needs following a disaster can lead to social fragmentation and disillusionment in survivors [61, 62].

Community cohesion can be defined as a bonding effect in communities who work together to overcome a common challenge or barrier for their collective wellbeing. Prior studies have found a positive correlation between cohesion and resilience [63, 64]. Community cohesion typically peaks during a honeymoon phase soon following the impact of the disaster and a heroic response phase from the community, as illustrated by the disaster collective reactions model [65, 66]. Over 60 respondents reported the desire to help others or asked how they could help their community recover. In the aftermath of a wildfire, communities may coalesce to meet the needs of those directly affected. This social network of neighbors, friends and relatives can be galvanized by wildfires and other disasters to take actions to help others [67]. However, helping other families created additional stress for some, as one family described how:

*We hosted [a] friend and her family and pets that lost their home. […] While grateful we could help others, the additional persons and pets significantly disrupted our normal routines (eating, sleeping, daily activities)—creating additional stress…*

Community members were often mentioned alongside household needs as a source of shelter, resources, and information. While many examples of community cohesion in our survey involved physical interactions, many households found fellowship, reassurance, and resources online. One household reported mental health-related needs, and when services were not available, *‘…join[ed] [a Facebook group] with over 60 K members. We all have the same stories and no one can get appointments because either counselors are booked for month, or do not take our insurance or are on prolonged vacations or are dealing with their own losses.’* Online community groups like the one above, whether formal and informal, represent an opportunity to researchers and agencies alike to provide targeted information and updates while also identifying outstanding needs. Nevertheless, in the face of wildfires as well as other community-wide disasters arising from climate change, expanding the workforce of individuals trained in counseling in order to meet the needs of those suffering from extreme stresses warrants highlighting and prioritization.

Addressing these complicated and related issues is beyond the scope of individual agencies, but require coordinated efforts across disaster response organizations to create a safety net capable of coordinating the many groups focused on disaster recovery [68]. The Federal Emergency Management Agency (FEMA) and the Substance Abuse and Mental Health Services Administration (SAMHSA) are instrumental in providing support and resources. The responses in this study highlight the value of recovery planning and communication. During a disaster, reliable information on available resources can be difficult to obtain, or be out-of-date by the time it reaches remote households, leading many households to rely on family, friends, neighbors and the internet for information. Of the 194 households that identified information needs, some reported that ‘It was hard to get specific and accurate information and misinformation abounded.’ Others, seeking information, said that ‘Since people were not in their homes or at work, our communication networks were compromised.’ Needs in recovering communities can include a lack of information or resources. While most respondents did not mention any specific organization, two—FEMA and CalFIRE—were identified as organizations that respondents expected to have wildfire-related information. Many responses in the week after the 2017 wildfires mentioned a need for earlier evacuation warnings, or more consistent guidance through the period immediately post-wildfire. Informational needs co-occur with many other physical needs, physical and mental health needs, and air-related needs. Interpersonal, material, organizational, environmental, and other general information is needed to resolve concerns such as those pertaining to insurance, housing, and communication. However, access to this information, particularly via the internet, raises an additional concern of equity in recovering communities. Higher income populations have been found to be more likely than lower income populations to seek information for health protection when exposed to wildfire smoke [69]. Internet access has been identified as a social determinant of health in many capacities, including by access to information and in community and social contexts [70]. Needs assessment instrument distribution can maximize participation by utilizing a wide variety of data collection platforms to effectively reach multicultural and non-English-speaking populations.

### 4.1. Disaster support

These findings highlight the disaster support gap between households with reported needs and the requisite disaster recovery resources. The documented information needs following the 2017 wildfires underscore the importance of communication and collaboration between disaster response and recovery agencies and impacted households. The gap between local expectations of federal disaster support and the implementation of recovery support activities can be overlooked by affected communities (i.e. local government, individual households) navigating comprehensive federal processes in the chaotic period following a disaster. Indeed, research by the Government Accountability Office [71] as well as Rosenthal *et al* [37] highlight disconnects between available federal disaster support and local government [37, 71]. Many respondents appeared to be confused as to the role of key agencies such as FEMA. Those who identified information needs expressed their lack of familiarity of roles of different agencies and the distinctions between the disaster response versus recovery support. Further public education about the roles and resources available from disaster support agencies is needed in this era of rolling crises stemming from climate change. Changing information-related needs identified by affected households may reflect organizational differences within the disaster response and recovery domain.

Local government plays an immediate role responding to disasters by providing support to impacted communities with police, fire, and medical aid and civil engagement. It also communicates needs on behalf of its constituents to state and federal organizations. Collaborative networks of local agencies such as NGOs, civic or volunteer groups, and faith-based organizations as well as individuals may coordinate to meet emerging needs [61]. When properly coordinated with federal and state agencies, resources for funding, information, and operational management can quickly be deployed to respond to local conditions [62]. While many emergency preparedness planning documents focus on the acute phase, academic interest in long-term community recovery is growing [67, 72–74]. Despite many persistent and ToS comments focusing on household-specific needs, other households raised broader concerns, such as:

*Certainty of continued employment with the County of Sonoma. The County is out nearly $25 million it spent on the fire and it is not certain this expenditure will be reimbursed by the State or the Federal Government. They may need to lay off employees at a time when we need all the help we can get in Sonoma County.*

Many of the responsibilities for recovery and continued operations fall to city or county agencies, which must maintain existing services in addition to disaster response [62]. They are a crucial facilitator for essential services among affected households. For instance, health departments serve the impacted communities and can maximize impact due to their familiarity with existing health resources (e.g. healthcare providers) and other supportive services [74].

### 4.2. Needs assessments during wildfires

Needs assessments can play a vital role in coordinating complex disaster response. The WHAT-Now needs assessment findings illustrate the ways in which some response efforts left needs unmet following the 2017 wildfires. For instance, our findings identify an opportunity for providing sustained mental health services immediately following wildfires such that mental health needs are attended to in the months to years following a disaster. Limited research exists on the collective, systematic proficiency of disaster recovery networks for responding to community needs. In addition to scholarly attention involving post-disaster monitoring and evaluation efforts, there is more work to be done to identify what needs are not being met by current public health and emergency preparedness systems. Conducting needs assessments in post-disaster communities can inform emergency preparedness and response organizations coordination by allowing agencies across a network to meet community needs as they arise, evolve, and persist over time.

More needs assessments in disaster impacted communities can provide research, public health, and government sectors with evidence to better understand what recovery and resource needs are not being met by the current systems, and to implement appropriate emergency preparedness and response improvements. Diversifying outreach strategies for needs assessment surveys can strengthen sampling methods and better reach vulnerable populations of interest. SAMHSA recommends materials distribution methods such as tabling at local community gathering spaces, distributing materials at local businesses, grocery stores, etc, and providing informational sheets translated into multiple languages to ensure cultural competence [65, 66]. However, in the wake of disasters including wildfires, these community spaces may be destroyed or damaged, and community members may have evacuated or been displaced, hampering efforts such as centralized information and other resources, as was the case in 2017. Telehealth has emerged as an effective method for providing medical and mental health resources in the wake of a disaster and other disruptions such as the COVID-19 pandemic. California HOPE Sonoma was set up after the fires to provide crisis counseling following the 2017 wildfires in Napa and Sonoma counties, with funding by FEMA and administrative support from the California State Mental Health Authority in conjunction with the Sonoma County Health Services. This program fielded thousands of calls each month, serving over 90 000 individuals over a three and a half year period following the wildfires [60]. Data collected in partnership with northern California universities, including UC Davis, successfully supported requests to extend California HOPE Sonoma twice, given how great and long-lasting the needs of wildfire-affected households were for mental health resources [58, 60].

As additional needs assessment studies emerge, there is more work to be done to link existing recovery efforts to the actual needs, particularly of those with fewer resources (non-homeowners, those with inadequate home insurance) and additionally to concrete metrics of recovery such as stable housing. Schnall *et al* note that ‘the majority [of rapid assessments] did not document implementation of recommendations based on [their] findings’—a crucial next step in understanding effective response and recovery strategies [75]. Incorporating monitoring and evaluation measures into needs assessments can track progress of needs being met in communities recovering from disasters and indicate the effectiveness of recommendations being made by needs assessments.

### 4.3. Limitations

There are some limitations of our study that merit consideration. Nearly 75% of respondents with reported needs came from Sonoma County, and due to the disruptive nature of these wildfires, it is likely that some respondents reported their current (displaced) addresses. Although the survey was quite explicit in stating that those who were ‘evacuated or displaced’ should report ‘their address as of 8 October 2017 even if the structure has been destroyed’. Furthermore, the survey accommodated those not willing to give an exact address with the option to provide an intersection within a 5 min walk from their home; a sizable proportion opted for this alternative, leaving very few with no location information at all [42]. Thus, our data reflects respondents from beyond the eight counties most directly affected by fire exposures, though respondents from these counties comprise over 95% of respondents with reported needs. In comparison with the demographics of Sonoma County and the other represented counties, the percentage of Hispanic respondents was lower than the general population. This likely occurred in part because data collection was through an online survey, which would not have reached those without internet access, regardless of having a Spanish version available. While certain demographics may have been more responsive to convenience sampling, we also implemented a door-to-door strategy to recruit a probability sample selected by scientific sampling to preferentially include neighborhoods with lower socioeconomic status. More than 70% of survey respondents were homeowners, and those who actually lost their homes included a disproportionately high number of wealthier neighborhoods (e.g. that were built in the WUI). Nevertheless, efforts to include renters’ perspectives could even the ‘property bias’ in disaster scholarship [33]. We observed non-specific language in respondents’ discussion of mental health related needs, which we coded as mental health impacts.

### 4.4. Preparing for the next wildfire

Our research identifies persistent, widespread needs that extend beyond a single county or community and point to broad post-fire barriers to stable long-term housing. Although our work captures needs in the months after a disaster, additional follow-up will be required to understand the long-term needs and impacts that can follow a wildfire. Addressing these ongoing needs for affected communities, and with particular attention to vulnerable populations, should be a high priority across local, state, and federal disaster response agencies. To do so may require workforce development in several areas, and our data shine the spotlight on the need for immediate and long-term mental health support services. A holistic approach to preparedness and recovery could help pair impacted households not only with available shelter, but also reliable mental health resources like trauma counseling and therapy. The analysis presented here provided only a snapshot at two time points, yet symptoms of mental health problems may not manifest until long after the disaster event, potentially a year or more, underscoring the need for longitudinal study of this cohort. Continued work is needed to better understand how household needs change over time and how to assess and address the needs reported above. Studies on disaster recovery in global settings have highlighted the importance of trustworthy public health messaging before and after wildfires, along with understanding what delivery methods are successful and needed by vulnerable groups [76–79]. Building rapport with communities to establish trust in the credibility and quality of shared information is necessary for local, state, and federal agencies to effectively communicate with at-risk communities in order to mitigate the wildfire impacts and associated mental stress [78].

Consistent monitoring and evaluation protocols are critical to understanding what communities need to recover and helping public health agencies to better identify effective recovery strategies. Building on existing survey tools such as SAMHSA’s can assist researchers identify which resources are being provided, and ensure they are reaching the households. Implementing monitoring and evaluation efforts are crucial to assess the effectiveness of existing recovery efforts, identify gaps, and provide evidence-based models so that disaster recovery agencies can adapt existing resources using proven and effective methodologies to meet the needs of future disaster-affected households. A possible solution would be an information and resource hub (physical and/or digital) for disaster-impacted communities. Centralizing and consolidating resources would provide current and consistent information to affected communities while offering a channel for agencies to respond to the emergent and dynamic needs. The efficient flow of information and resources would make these hubs a logical setting for monitoring and evaluation efforts.

## 5. Conclusion

This study provides insight into household needs following 2017 Northern California Wildfires. More than one-third of those with needs had physical needs either persistently or just at the ToS. The highest specific needs within that group were housing and financial needs. Key findings also reflect all four broad categories: physical needs, air quality, health—both physical and mental—and information. In the week after the outbreak of the wildfires, the most commonly reported needs were: better air quality, basic needs, utilities, and housing, whereas at the time of the survey (months later), housing, financial and mental health needs were the greatest needs. In addition to detailing household needs and how those needs change over time, we also introduce a key distinction: needs vs. impacts. While health-related sequalae following wildfires can inform health and emergency services providers, there is potential for impact-related interventions. These include addressing persistent unmet housing needs, a byproduct of broader housing availability constraints but vastly exacerbated by the wildfires, as well as the mental impacts that restrict both short and long-term community recovery capacity. Information needs were also prominently represented in our data, with households stressed about housing insecurity, how to navigate insurance companies and the obstacles to getting sufficient money to return to their prior standard of living. These needs represent gaps within disaster recovery resources and present an opportunity to better communicate the roles, responsibilities, and capabilities of involved agencies to the affected public. As communities continue to contend with wildfire disasters, responding agencies and experts can develop strategies to meet communities’ needs by conducting needs assessments with sustained monitoring and evaluation that integrate collaboration across jurisdictions.

## Data Availability

The data cannot be made publicly available upon publication because they contain sensitive personal information. The data that support the findings of this study are available upon reasonable request from the authors.
